# A longitudinal cohort study: developing an interpretable machine learning model to predict incident depression risk in elderly Chinese patients with gastrointestinal or chronic liver diseases

**DOI:** 10.1186/s12877-026-07239-7

**Published:** 2026-02-25

**Authors:** Yin Chen, Mingyu Chen

**Affiliations:** 1 Department of General Surgery, The Affiliated Xuancheng Hospital of Wannan Medical College (Xuancheng People’s Hospital), Xuancheng, 242000 P. R. China; 2 Department of Internal Medicine, Guangwai Hospital (Guangwai Geriatric Hospital) of Xicheng District, No. 2A, Sanyili, Xicheng District, Beijing, 100053 P. R. China

**Keywords:** Depression, Machine learning, CHARLS, Shapley additive explanation, Gastrointestinal diseases, Chronic liver diseases

## Abstract

**Background:**

Depression is highly prevalent in elderly patients with gastrointestinal (GID) or chronic liver diseases (CLD), significantly impairing quality of life and treatment outcomes. This study aimed to develop and validate an interpretable machine learning (ML) model to identify depression risk in this population, overcoming the “black box” limitation of conventional ML.

**Materials and methods:**

This prospective analysis utilized data from the baseline (2018) and follow-up (2020) waves of the China Health and Retirement Longitudinal Study (CHARLS). Potential predictors measured at baseline were selected via Least Absolute Shrinkage and Selection Operator (LASSO) regression. The outcome was incident depression at the 2020 follow-up, defined by a CES-D-10 score ≥ 10 among participants free of depression at baseline. Ten ML algorithms were employed to construct models. Performance was evaluated using the area under the receiver operating characteristic curve (AUC), sensitivity, specificity, precision, F1-score, calibration curves, and decision curve analysis. The SHapley Additive exPlanations (SHAP) framework interpreted feature contributions.

**Results:**

Among 1,353 participants (424 with depression), LASSO identified 10 key predictors. The Logistic Regression (LR) model demonstrated optimal discriminative performance, with an AUC of 0.723 (95% CI: 0.674–0.772). SHAP analysis revealed the top five predictors: self-reported health, life satisfaction, gender, education, and memory scores.

**Conclusions:**

We developed an interpretable ML model for predicting depression risk in elderly patients with GID or CLD. This tool aids early detection and intervention, potentially improving clinical outcomes in this vulnerable population.

**Supplementary Information:**

The online version contains supplementary material available at 10.1186/s12877-026-07239-7.

## Introduction

Gastrointestinal diseases (GID) and chronic liver diseases (CLD) constitute a range of highly prevalent digestive system disorders, both of which are associated with increased mortality risk and substantial healthcare expenditures [1]. GID, including conditions such as gastritis, irritable bowel syndrome (IBS), functional dyspepsia (FD), peptic ulcer disease (PUD) and gastroesophageal reflux disease (GERD), represents a significant worldwide health challenge. In addition to reducing patients’ quality of life, GID imposes a substantial financial strain on healthcare systems globally [2]. In 2019, digestive diseases accounted for approximately 2.86 billion cases globally, causing 8 million fatalities and 277 million disability-adjusted life years (DALYs) lost [3]. The growing burden of these conditions is driven by multiple factors, including age-related physiological decline, lifestyle choices, dietary habits, and long-term exposure to environmental and genetic risks [3]. Among older adults, GID has wide-ranging effects. From a public health perspective, it significantly increases healthcare utilization, leading to higher rates of hospital admissions, outpatient consultations, and prolonged medication use [4]. Socially, GID contributes to isolation, reduced productivity, and increased reliance on caregivers, compounding the psychological distress experienced by patients [4]. Prevalence rates of GID are exceptionally high in older populations [5, 6]. Chronic liver diseases (CLD), including steatohepatitis and various forms of hepatitis, also pose a significant global public health challenge and serve as substantial contributors to worldwide illness and fatality. An estimated 1.6 billion people are affected by CLD globally [7, 8], including roughly 20% of China’s population [9, 10]. In 2018, CLD complications such as cirrhosis were responsible for approximately 1.32 million deaths worldwide, comprising 2.4% of total global mortality. Beyond reducing quality of life, CLD imposes considerable economic costs on healthcare systems [11]. These compelling figures underscore the urgent need for effective prevention and intervention strategies that address both GID and CLD.

Notably, the burden of GID and CLD extends beyond physical impairment, affecting the digestive system for GID and the liver for CLD, to include mental health impacts. Broad epidemiological evidence indicates that depressive symptoms are significantly more common in individuals with GID or CLD compared to the general population. For example, prior research has shown that *Helicobacter pylori* infection, a well-established major etiological factor for PUD [12], significantly increases the risk of depressive symptoms [13]. And patients with FD or IBS, two common GID, show a notably higher risk of co-occurring depression. Studies report depression rates of 20.9% in FD and 23.3% in IBS cases [14, 15], figures that are roughly 1.5–2 times greater than those in the general population [16]. Additionally, evidence suggests that some individuals with CLD also experience more severe depressive symptoms [17].

Depression, a prevalent mental health condition, is significantly correlated with elevated risks of disability and mortality, especially in the elderly population. Worldwide, depression impacts over 300 million people, with the highest prevalence observed among adults aged 55 to 74 years, reaching 7.5% in women and 5.5% in men [18]. China is undergoing rapid demographic aging, and its elderly population is expected to reach 440 million by 2050 [19]. This demographic transition poses substantial public health challenges, notably through its role in elevating already rising rates of depression among older adults [18]. Moreover, studies have highlighted that the increasing incidence of depression in elderly Chinese individuals with GID or CLD poses a pressing challenge to the national healthcare system [20]. Age-related physiological changes compound this challenge by potentially disrupting gastrointestinal and hepatic function.

Meanwhile, depressive symptoms can accelerate the progression of GID and CLD, creating a vicious cycle that ultimately impairs patients’ quality of life and may even threaten their survival [21–24]. Consequently, implementing proactive mental health interventions, focused on early identification and treatment, for elderly patients with GID or CLD is critical to improving their overall health outcomes. However, the substantial financial burden of large-scale epidemiological studies limits their practicality.

In current clinical settings, depression risk assessment depends heavily on clinicians’ subjective judgment due to the scarcity of objective, quantifiable tools. Effectively and promptly identifying depression risk in high-risk groups remains challenging. Recent advances in machine learning (ML) offer promising alternatives, however. ML techniques can identify key features from large datasets and support the development of predictive models, improving the accuracy of risk stratification and enabling earlier intervention [25]. A major obstacle to clinical adoption, however, is the “black box” nature of conventional ML models [26]. Interpretable ML, an emerging subfield, addresses this issue through frameworks such as the Shapley Additive exPlanations (SHAP), which originated from cooperative game theory —a field receiving growing emphasis —to enhance model interpretability and accountability [27]. By integrating SHAP, interpretable ML enhances the transparency of traditional models. SHAP quantifies the contribution of individual input features, helping to identify driving factors behind depression risk in older adults with GID or CLD. Furthermore, SHAP offers intuitive visualization tools, including summary, dependency, and force plots, that translate complex model outputs into clinically actionable insights, thereby facilitating the application of ML-derived findings in real-world practice.

In line with this methodological shift, the present study utilized data from the 2018–2020 waves of the China Health and Retirement Longitudinal Study (CHARLS) to construct an interpretable ML model for predicting depression risk among older adults with GID or CLD. Our aims were to identify the factors most strongly associated with depression in this population and to evaluate the performance of multiple ML algorithms. This approach builds upon a growing body of research applying interpretable ML frameworks to CHARLS data for health outcome prediction. For instance, a recent longitudinal CHARLS‑based study used an explainable ML framework to predict 10‑year cognitive impairment risk in early‑stage cardiovascular‑kidney‑metabolic syndrome, highlighting the value of interpretable models for risk stratification in complex chronic diseases [28]. Following a similar interpretable framework, our study seeks to develop a tailored predictive tool for depression risk, with the ultimate goal of supporting early detection and improving health outcomes and quality of life in this high-need geriatric group.

## Methods

### Study Design and Participants

This study utilized data from the China Health and Retirement Longitudinal Study (CHARLS), a nationally representative longitudinal survey of middle-aged and older adults in China aged 45 and above. CHARLS covers extensive information on demographic characteristics, health status, health service utilization, and associated expenditures. To date, five waves of data have been made publicly available (2011–2012, 2013, 2015, 2018, and 2020), with a large sample size ensuring national representativeness [6]. The Institutional Review Board at Peking University granted approval for the study protocol in line with the Declaration of Helsinki (Approval No. IRB00001052-11015) before the commencement of participant recruitment.

Clinical trial number: not applicable.

**Fig. 1:**
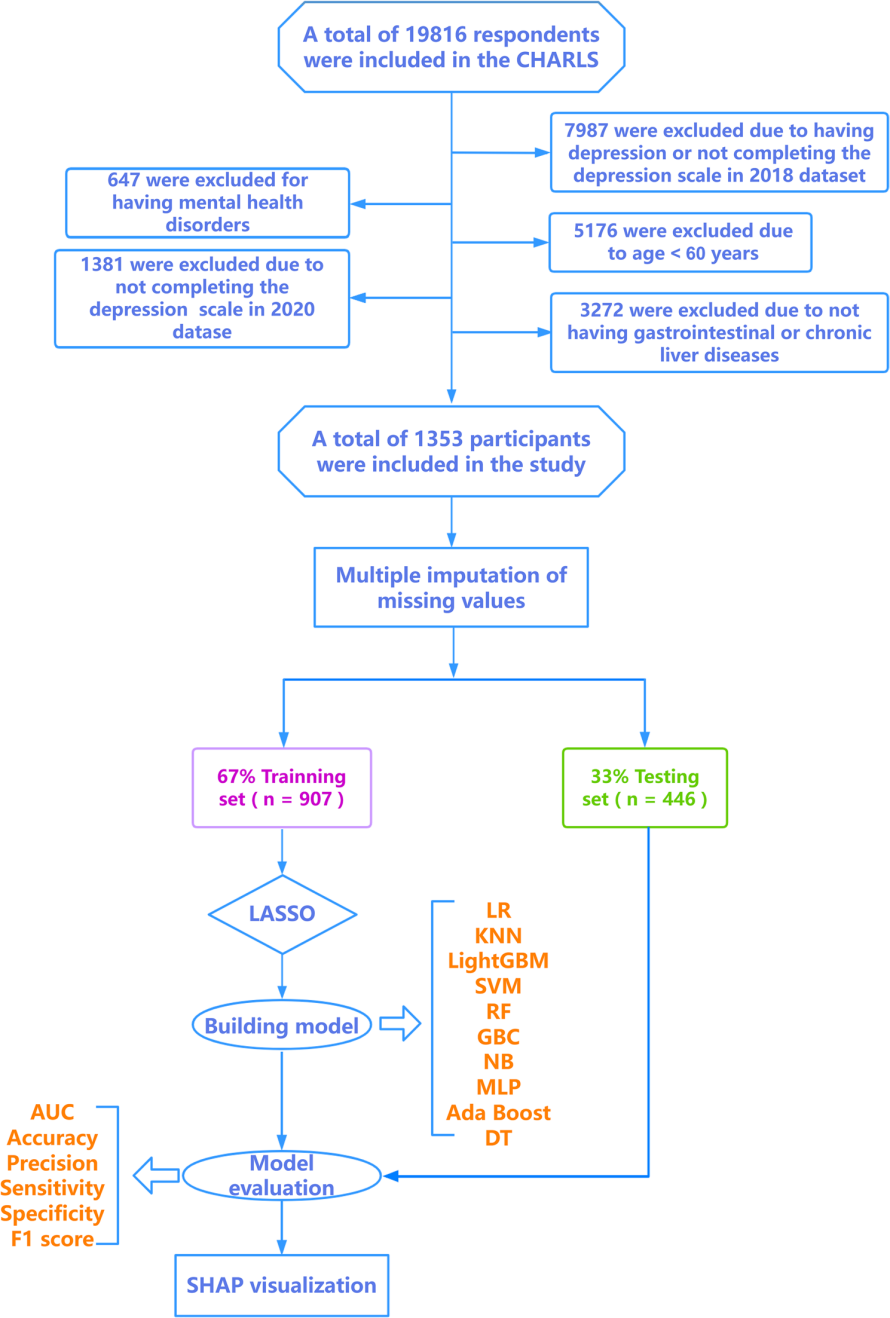
Flow chart of this study. LR, Logistic Regression; MLP, Multilayer Perceptron; KNN, k-Nearest Neighbors; NB, Naive Bayes; GBC, Gradient Boosting Classifier; LightGBM, Light Gradient Boosting Machine; Ada Boost, Adaptive Boosting; SVM, Support Vector Machine; RF, Random Forest; DT, Decision Tree; LASSO, Least Absolute Shrinkage and Selection Operator

This was a prospective cohort study leveraging the longitudinal structure of CHARLS. The study population consisted of participants who were free of depression at the baseline (2018) assessment but had a physician-confirmed diagnosis of GID or CLD. These individuals were then followed for the incident outcome of depression at the subsequent wave (2020). Specifically, inclusion criteria were: (1) aged 60 years or above; (2) clinical diagnosis of either GID or CLD (confirmed via self-report of physician diagnosis). Exclusion criteria comprised: (1) baseline depressive symptoms (2018), a history of mental health disorders, or failure to complete the depression scale in 2018; (2) inability to independently provide information; (3) non-participation in the 2020 follow-up survey; (4) failure to complete the depression scale in 2020. This design ensured that predictors were assessed prior to the occurrence of the outcome, establishing a temporal sequence essential for predictive modeling of incident events. The final analytical sample consisted of 1,353 elderly participants. A detailed flowchart of participant selection is provided in Fig. 1.

### Assessment of depression

Depression Scale (CES-D-10) to evaluate depressive symptoms among participants. This widely adopted tool assesses the frequency of various mood disturbances, such as sadness and hopelessness, over the preceding week. Responses were collected using a four-point scale: 0 indicated rare or no occurrence (less than 2 days), 1 represented occasional presence (2 days), two denoted frequent occurrences (3–4 days), and 3 signified persistent symptoms (5–7 days). Total scores range from 0 to 30, with higher values reflecting more severe depression [29]. Previous research has demonstrated that the CES-D-10 exhibits strong reliability and validity for depression screening in older Chinese populations [30]. Based on prior research, a cutoff score of 10 is recommended for defining depression in the CHARLS population [31]. In this study, we defined depression as a score of 10 or higher, and scores below 10 as non-depression. This binary classification (depression vs. non-depression) served as the outcome variable for all analytical models.

### Clinical outcome

The primary outcome of this study was incident depression, defined as the new onset of depressive symptoms during the follow-up period. Operationally, a participant was classified as having incident depression if they met the following criteria: (1) were classified as non-depressed (CES-D-10 < 10) at the baseline (2018) assessment, and (2) were classified as depressed (CES-D-10 ≥ 10) at the follow-up (2020) assessment. This approach aligns with established longitudinal methodologies for studying disease incidence and minimizes the bias from pre-existing conditions.

### Assessment of gastrointestinal disease and chronic liver disease

We identified cases of GID and CLD using responses from health status questionnaires administered during the 2018 baseline survey. Participants reported whether a physician had diagnosed them with a digestive disease (excluding tumors or cancers) or a liver disease (excluding fatty liver, tumors, or cancers). We classified respondents answering “yes” to the digestive disease question as having GID, and those affirming a liver disease diagnosis as having CLD.

### Assessment of covariates

We obtained demographic and lifestyle information through in-person interviews. The data included age, gender (male or female), marital status (married/cohabited or unmarried), ethnicity (Han or non-Han), and residential area (rural or urban). Educational background was categorized as below elementary school, elementary school graduate, middle school graduate, high school graduate and above. Family characteristics encompassed family size, number of surviving children, parents’ economic support for children, children’s economic support for parents, health insurance, retire and pension. The recorded healthcare utilization metrics included outpatient visits and the number of outpatient visits in the past month, hospitalizations in the past year, the number of hospitalizations, and the length of hospital stay (LOS) in the past year. Smoking and drinking behaviors were classified as never, ever or current. Physical exercise and social activity engagement were each dichotomized into two categories: yes or no.

Participants provided self-reports of physician-diagnosed chronic conditions, such as dyslipidemia, cancer, heart disease, hypertension, stroke, lung disease, mental illness, arthritis or rheumatism, kidney disease, asthma, and memory-related disorders (e.g., Alzheimer’s disease, Parkinson’s disease, and cerebral atrophy). The total number of chronic diseases was categorized into four groups: 1, 2, 3, and 4 or more. We assessed physical function using two established instruments: the Activities of Daily Living (ADL) scale and the Instrumental Activities of Daily Living (IADL) scale [32]. Meanwhile, chronic pain was defined as pain that either persists beyond the anticipated healing period or continues even when there is no underlying tissue damage [33]. Other physical condition indicators included edentulism (complete tooth loss).

Mental health-related factors included three key dimensions: hope for the future, life satisfaction, and self-reported health. Specifically, hope for the future was categorized into two responses: “no” and “yes.” Both life satisfaction and self-reported health were assessed using a three-level classification system, with options of “good,” “fair,” and “poor” for each [34]. Memory function was assessed using an immediate recall test. Participants were administered 10 words and asked to recall them immediately after presentation [35]. Sleep-related variables were also assessed using a self-reported questionnaire. Drawing on findings from previous studies [36–38], a daily sleep duration of 6–8 h was defined as “normal” for Chinese adults aged 40 years and older. Accordingly, we classified sleep duration into three categories: those with short sleep (fewer than 6 h), normal sleep (6 to 8 h), or long sleep (8 h or longer). Sleep quality was rated as “good,” “fair,” or “poor” based on participants’ self-reported assessments.

### Missing value processing

Missing data are common in longitudinal surveys such as CHARLS. To preserve sample size and minimize selection bias, we implemented a multiple imputation strategy using the Multivariate Imputation by Chained Equations (MICE) algorithm via the mice package in R. Variables with missing rates exceeding 30% were excluded prior to imputation to avoid introducing excessive uncertainty. The remaining variables with missing values were imputed iteratively across five independent datasets, incorporating all analysis variables including the outcome to maintain consistency in the imputation model. To prevent information leakage between training and testing sets, all imputation procedures were performed prior to data splitting. After generating five complete imputed datasets, each dataset was randomly partitioned into a training subset (67%) and a testing subset (33%) using the same random seed to ensure consistent partitioning across imputations. Model development and hyperparameter tuning were conducted separately within each imputed training set, and performance was evaluated on the corresponding imputed test set. Final performance metrics were pooled across the five imputed datasets using Rubin’s rules to obtain robust estimates of model discrimination, calibration, and clinical utility. The distribution of missing data before imputation is illustrated in Supplementary Fig. 1. This approach ensures that the imputation process does not introduce bias from the test set into training, thereby maintaining the validity of model evaluation and enhancing the reproducibility of our analytical pipeline.

### Feature selection

We performed feature selection using the Least Absolute Shrinkage and Selection Operator (LASSO) method. LASSO achieves variable selection by introducing an L1 penalty term into the least squares loss function, which forces the coefficients of certain predictors toward zero and effectively removes them from the model. A key parameter in LASSO is λ (λ ≥ 0), which controls the strength of regularization. When λ equals 0, the model reduces to ordinary least squares regression. To determine the optimal λ value, we employed 10‑fold cross‑validation. The dataset was randomly partitioned into 10 non‑overlapping subsets. In each iteration, nine subsets were used as the training set to estimate model coefficients across a range of λ values, and the remaining subset served as the test set for prediction. This process was repeated 10 times so that each subset was used once as the test set. The optimal λ was selected based on the minimum mean squared error (MSE) averaged over the 10 prediction rounds. Two commonly used criteria for choosing λ were considered: λ.min, which corresponds to the λ yielding the lowest cross‑validation error, and λ.1se, which represents the largest λ within one standard error of the minimum error, thereby providing a more parsimonious model. The choice between these depends on the study context and objectives. LASSO also helps address multicollinearity among predictors by driving coefficients of redundant or irrelevant variables to zero, thereby enhancing model stability and interpretability [39]. Importantly, all feature selection steps were conducted exclusively within the training set. The selected feature subset was then fixed and applied directly to the independent test set for validation without any further selection or refitting, ensuring an unbiased evaluation of model generalizability.

To further mitigate potential multicollinearity among the retained variables, we calculated variance inflation factors (VIFs) for all selected features [40]. A conservative threshold of VIF < 5 was applied to ensure acceptable independence among predictors, consistent with established standards for high‑dimensional epidemiological modeling. Only features meeting this criterion were carried forward into subsequent model training and validation stages.

### Model construction and evaluation

ML approaches are broadly divided into four primary categories: supervised, unsupervised, semi-supervised, and reinforcement learning. This study focuses on assigning participants to two classes (depression vs. non-depression), which constitutes a binary classification task. Thus, supervised learning algorithms were deemed the most appropriate approach [41]. We utilized ten commonly used supervised learning algorithms to construct predictive models: Logistic Regression (LR), Multilayer Perceptron (MLP), k-Nearest Neighbors (KNN), Naive Bayes (NB), Gradient Boosting Classifier (GBC), Light Gradient Boosting Machine (LightGBM), Adaptive Boosting Classifier (AdaBoost), Support Vector Machine (SVM), Random Forest (RF), and Decision Tree (DT).

We randomly allocated the data into a training subset (67%) for building the model and a testing subset (33%) for validation, enabling a robust evaluation of predictive performance and generalizability. Furthermore, to mitigate class imbalance in the binary outcome (depression vs. non-depression), we applied the Synthetic Minority Oversampling Technique (SMOTE). Hyperparameters for all models were optimized using a grid‑search strategy combined with 10‑fold cross‑validation, which enhanced robustness and reduced the risk of overfitting.

Model performance was evaluated along three core dimensions: discriminative ability, calibration ability, and clinical utility. Discrimination, which reflects how well the model separates outcome classes, was quantified using metrics including the area under the receiver operating characteristic (ROC) curve (AUC), accuracy, sensitivity, specificity, precision, and the F1-score. Calibration was evaluated by comparing predicted probabilities against observed outcomes through calibration curves and the Brier score. Clinical value was measured using decision curve analysis (DCA), which estimates the net benefit of each model across various risk thresholds. Additionally, pairwise DeLong tests were conducted to statistically compare AUCs across all ten algorithms. Together, these complementary metrics provided a comprehensive evaluation of both statistical performance and potential clinical applicability.

### Evaluation of the importance of variables

To evaluate predictor importance, we applied the SHAP framework, a game theory-based approach for interpreting ML outputs [42]. SHAP values quantify the influence of individual features on the model’s predictions at the level of each observation. For the present study’s outcome, positive SHAP values signify an elevated risk of depression, whereas negative SHAP values denote a reduced risk of the condition. This framework enhances the interpretability of the ML model, addressing the “black box” limitation of traditional algorithms.

### Statistical analysis

Continuous variables are presented as mean ± standard deviation (SD), while categorical variables are summarized as frequency (n) and percentage (%). We compared continuous measures between groups using independent samples t-tests for normally distributed data; the Mann-Whitney U test was applied for non-normally distributed variables. Categorical variables were analyzed using the Chi-square test. All binary variables were coded as 1 (“yes”) or 0 (“no”). All statistical analyses were performed using R (http://www.R-project.org; The R Foundation) and Free Statistics software version 2.1. A two-sided p-value below 0.05 indicated statistical significance.

## Results

### Subject characteristics

The ML prediction models were developed using a total of 1,353 older adults with GID or CLD who were free of depression at baseline. Over the two-year follow-up period from 2018 to 2020, 424 individuals (31.34%) developed incident depression (Table 1). Depressed individuals showed significantly different characteristics compared to those without depression: they tended to be older, female, unmarried, or residing in rural areas; had lower educational attainment (below elementary level), lacked health insurance, provided less financial support to their children, and had more surviving children. Additionally, they were more likely to have never smoked, have a history of alcohol use, not participate in social activities, not be retired, have shorter sleep duration, report fair sleep quality, experience chronic pain, self-report poor health status, have low life satisfaction, express uncertainty about the future, exhibit lower memory scores, and have higher ADL and IADL scores. They also had higher rates of disability, falls, hip fractures, edentulism, and four or more chronic comorbidities.

**Table 1:** Baseline characteristics of the participants

Variables	Total (*n* = 1353)	No depression (*n* = 929)	Depression (*n* = 424)		*p*-Value
**Age (years)**,** Mean ± SD**	67.8 ± 5.8	67.5 ± 5.6		68.3 ± 6.1	0.028
**Gender**,** n (%)**					< 0.001
Female	594 (43.9)	353 (38)		241 (56.8)	
Male	759 (56.1)	576 (62)		183 (43.2)	
**Ethnicity**,** n (%)**					0.556
Non-Han	81 (6.0)	58 (6.2)		23 (5.4)	
Han	1272 (94.0)	871 (93.8)		401 (94.6)	
**Marital status**,** n (%)**					0.018
Unmarried	206 (15.2)	127 (13.7)		79 (18.6)	
Married/Cohabitated	1147 (84.8)	802 (86.3)		345 (81.4)	
**Area of residence**,** n (%)**					< 0.001
Urban	586 (43.3)	436 (46.9)		150 (35.4)	
Rural	767 (56.7)	493 (53.1)		274 (64.6)	
**Education level**,** n (%)**					< 0.001
Below elementary school	530 (39.2)	302 (32.5)		228 (53.8)	
Elementary school graduate	359 (26.5)	263 (28.3)		96 (22.6)	
Middle school graduate	287 (21.2)	225 (24.2)		62 (14.6)	
High school graduate and above	177 (13.1)	139 (15)		38 (9)	
**Insurance**,** n (%)**					0.047
None	29 (2.1)	15 (1.6)		14 (3.3)	
Yes	1324 (97.9)	914 (98.4)		410 (96.7)	
**Cfsfp**,** Median (IQR)**	2750.0 (700.0, 7000.0)	3000.0 (600.0, 7000.0)		2500.0 (947.5, 6000.0)	0.575
**Pfsfc**,** Median (IQR)**	0.0 (0.0, 1200.0)	0.0 (0.0, 2000.0)		0.0 (0.0, 600.0)	< 0.001
**Family size**,** Mean ± SD**	2.7 ± 1.5	2.7 ± 1.4		2.7 ± 1.5	0.78
**Number of Surviving Children**,** Mean ± SD**	2.8 ± 1.3	2.7 ± 1.3		3.0 ± 1.3	< 0.001
**Outpatient Visit**,** n (%)**					0.638
No	1117 (82.6)	770 (82.9)		347 (81.8)	
Yes	236 (17.4)	159 (17.1)		77 (18.2)	
**Number of Outpatient Visits**,** Median (IQR)**	0.0 (0.0, 0.0)	0.0 (0.0, 0.0)		0.0 (0.0, 0.0)	0.669
**Hospitalization**,** n (%)**					0.216
No	1077 (79.6)	748 (80.5)		329 (77.6)	
Yes	276 (20.4)	181 (19.5)		95 (22.4)	
**Number of Hospitalizations**,** Median (IQR)**	0.0 (0.0, 0.0)	0.0 (0.0, 0.0)		0.0 (0.0, 0.0)	0.264
**LOS**,** Median (IQR)**	0.0 (0.0, 0.0)	0.0 (0.0, 0.0)		0.0 (0.0, 0.0)	0.107
**Smoking**,** n (%)**					< 0.001
Never	676 (50.0)	426 (45.9)		250 (59)	
Ever	299 (22.1)	225 (24.2)		74 (17.5)	
Current	378 (27.9)	278 (29.9)		100 (23.6)	
**Drinking**,** n (%)**					< 0.001
Never	636 (47.0)	409 (44)		227 (53.5)	
Ever	234 (17.3)	157 (16.9)		77 (18.2)	
Current	483 (35.7)	363 (39.1)		120 (28.3)	
**Retire**,** n (%)**					< 0.001
No	977 (72.2)	630 (67.8)		347 (81.8)	
Yes	376 (27.8)	299 (32.2)		77 (18.2)	
**Social activity**,** n (%)**					0.001
None	621 (45.9)	399 (42.9)		222 (52.4)	
Yes	732 (54.1)	530 (57.1)		202 (47.6)	
**Exercise**,** n (%)**					0.207
No	106 ( 7.8)	67 (7.2)		39 (9.2)	
Yes	1247 (92.2)	862 (92.8)		385 (90.8)	
**Sleep duration**,** n (%)**					< 0.001
Short sleep	459 (33.9)	273 (29.4)		186 (43.9)	
Normal sleep	581 (42.9)	426 (45.9)		155 (36.6)	
Long sleep	313 (23.1)	230 (24.8)		83 (19.6)	
**Sleep quality**,** n (%)**					< 0.001
Good	775 (57.3)	570 (61.4)		205 (48.3)	
Fair	397 (29.3)	251 (27)		146 (34.4)	
Poor	181 (13.4)	108 (11.6)		73 (17.2)	
**Chronic pain**,** n (%)**					< 0.001
No	500 (37.0)	390 (42)		110 (25.9)	
Yes	853 (63.0)	539 (58)		314 (74.1)	
**Self-reported health**,** n (%)**					< 0.001
Good	269 (19.9)	222 (23.9)		47 (11.1)	
Fair	753 (55.7)	528 (56.8)		225 (53.1)	
Poor	331 (24.5)	179 (19.3)		152 (35.8)	
**Life satisfaction**,** n (%)**					0.008
Good	552 (40.8)	398 (42.8)		154 (36.3)	
Fair	755 (55.8)	507 (54.6)		248 (58.5)	
Poor	46 (3.4)	24 (2.6)		22 (5.2)	
**Hope**,** n (%)**					0.001
No	709 (52.4)	459 (49.4)		250 (59)	
Yes	644 (47.6)	470 (50.6)		174 (41)	
**Memory scores**,** Mean ± SD**	3.6 ± 2.0	3.8 ± 2.0		3.0 ± 1.9	< 0.001
**ADL scores**,** Median (IQR)**	0.0 (0.0, 0.0)	0.0 (0.0, 0.0)		0.0 (0.0, 1.0)	< 0.001
**IADL scores**,** Mean ± SD**	0.3 ± 0.8	0.2 ± 0.6		0.5 ± 1.0	< 0.001
**Disability**,** n (%)**					0.004
No	817 (60.4)	585 (63)		232 (54.7)	
Yes	536 (39.6)	344 (37)		192 (45.3)	
**Fall down**,** n (%)**					< 0.001
No	1116 (82.5)	789 (84.9)		327 (77.1)	
Yes	237 (17.5)	140 (15.1)		97 (22.9)	
**Hip fracture**,** n (%)**					0.023
No	1338 (98.9)	923 (99.4)		415 (97.9)	
Yes	15 (1.1)	6 (0.6)		9 (2.1)	
**Edentulism**,** n (%)**					0.014
No	1081 (79.9)	759 (81.7)		322 (75.9)	
Yes	272 (20.1)	170 (18.3)		102 (24.1)	
**The total number of chronic diseases**,** n (%)**					< 0.001
1	189 (14.0)	148 (15.9)		41 (9.7)	
2	331 (24.5)	237 (25.5)		94 (22.2)	
3	283 (20.9)	197 (21.2)		86 (20.3)	
≥4	550 (40.7)	347 (37.4)		203 (47.9)	

*Cfsfp* Children’s financial support for parents, *Pfsfc* Parents’ financial support for their children, *LOS* Length of Hospital Stay, *IADL* Instrumental Activities of Daily Living, *ADL* Activity of Daily Living

Additionally, we compared the baseline characteristics of participants between the training and testing sets; the full results are presented in Table 2. All baseline characteristics were well balanced between the two groups (all *P* > 0.05), providing a robust foundation for subsequent data analyses and interpretations.

**Table 2:** Comparison of variables between the training set and the testing set of elderly patients with gastrointestinal diseases or chronic liver diseases

Variables	Total (*n* = 1353)	Training set (*n* = 907)	Testing set (*n* = 446)		*p*-Value
**Age (years)**,** Mean ± SD**	67.8 ± 5.8	67.7 ± 5.6		67.8 ± 6.0	0.722
**Gender**,** n (%)**					0.470
Female	594 (43.9)	392 (43.2)		202 (45.3)	
Male	759 (56.1)	515 (56.8)		244 (54.7)	
**Ethnicity**,** n (%)**					0.864
Non-Han	81 (6.0)	55 (6.1)		26 (5.8)	
Han	1272 (94.0)	852 (93.9)		420 (94.2)	
**Marital status**,** n (%)**					0.759
Unmarried	206 (15.2)	140 (15.4)		66 (14.8)	
Married/Cohabitated	1147 (84.8)	767 (84.6)		380 (85.2)	
**Area of residence**,** n (%)**					0.741
Urban	586 (43.3)	390 (43)		196 (43.9)	
Rural	767 (56.7)	517 (57)		250 (56.1)	
**Education level**,** n (%)**					0.740
Below elementary school	530 (39.2)	351 (38.7)		179 (40.1)	
Elementary school graduate	359 (26.5)	240 (26.5)		119 (26.7)	
Middle school graduate	287 (21.2)	200 (22.1)		87 (19.5)	
High school graduate and above	177 (13.1)	116 (12.8)		61 (13.7)	
**Insurance**,** n (%)**					0.533
None	29 (2.1)	21 (2.3)		8 (1.8)	
Yes	1324 (97.9)	886 (97.7)		438 (98.2)	
**Cfsfp**,** Median (IQR)**	2750.0 (700.0, 7000.0)	2900.0 (775.0, 7000.0)		2500.0 (500.0, 6000.0)	0.220
**Pfsfc**,** Median (IQR)**	0.0 (0.0, 1200.0)	0.0 (0.0, 1325.0)		0.0 (0.0, 1000.0)	0.370
**Family size**,** Mean ± SD**	2.7 ± 1.5	2.6 ± 1.4		2.8 ± 1.5	0.065
**Number of Surviving Children**,** Mean ± SD**	2.8 ± 1.3	2.8 ± 1.3		2.8 ± 1.3	0.256
**Outpatient Visit**,** n (%)**					0.428
No	1117 (82.6)	754 (83.1)		363 (81.4)	
Yes	236 (17.4)	153 (16.9)		83 (18.6)	
**Number of Outpatient Visits**,** Median (IQR)**	0.0 (0.0, 0.0)	0.0 (0.0, 0.0)		0.0 (0.0, 0.0)	0.504
**Hospitalization**,** n (%)**					0.250
No	1077 (79.6)	730 (80.5)		347 (77.8)	
Yes	276 (20.4)	177 (19.5)		99 (22.2)	
**Number of Hospitalizations**,** Median (IQR)**	0.0 (0.0, 0.0)	0.0 (0.0, 0.0)		0.0 (0.0, 0.0)	0.247
**LOS**,** Median (IQR)**	0.0 (0.0, 0.0)	0.0 (0.0, 0.0)		0.0 (0.0, 0.0)	0.222
**Smoking**,** n (%)**					0.640
Never	676 (50.0)	445 (49.1)		231 (51.8)	
Ever	299 (22.1)	204 (22.5)		95 (21.3)	
Current	378 (27.9)	258 (28.4)		120 (26.9)	
**Drinking**,** n (%)**					0.640
Never	676 (50.0)	445 (49.1)		231 (51.8)	
Ever	299 (22.1)	204 (22.5)		95 (21.3)	
Current	378 (27.9)	258 (28.4)		120 (26.9)	
**Retire**,** n (%)**					0.903
No	977 (72.2)	654 (72.1)		323 (72.4)	
Yes	376 (27.8)	253 (27.9)		123 (27.6)	
**Social activity**,** n (%)**					0.281
None	621 (45.9)	407 (44.9)		214 (48)	
Yes	732 (54.1)	500 (55.1)		232 (52)	
**Exercise**,** n (%)**					0.820
No	106 ( 7.8)	70 (7.7)		36 (8.1)	
Yes	1247 (92.2)	837 (92.3)		410 (91.9)	
**Sleep duration**,** n (%)**					0.458
Short sleep	459 (33.9)	300 (33.1)		159 (35.7)	
Normal sleep	581 (42.9)	400 (44.1)		181 (40.6)	
Long sleep	313 (23.1)	207 (22.8)		106 (23.8)	
**Sleep quality**,** n (%)**					0.367
Good	775 (57.3)	529 (58.3)		246 (55.2)	
Fair	397 (29.3)	255 (28.1)		142 (31.8)	
Poor	181 (13.4)	123 (13.6)		58 (13)	
**Chronic pain**,** n (%)**					0.736
No	500 (37.0)	338 (37.3)		162 (36.3)	
Yes	853 (63.0)	569 (62.7)		284 (63.7)	
**Self-reported health**,** n (%)**					0.145
Good	269 (19.9)	193 (21.3)		76 (17)	
Fair	753 (55.7)	491 (54.1)		262 (58.7)	
Poor	331 (24.5)	223 (24.6)		108 (24.2)	
**Life satisfaction**,** n (%)**					0.501
Good	552 (40.8)	380 (41.9)		172 (38.6)	
Fair	755 (55.8)	497 (54.8)		258 (57.8)	
Poor	46 (3.4)	46 (3.4)		30 (3.3)	
**Hope**,** n (%)**					0.372
No	709 (52.4)	483 (53.3)		226 (50.7)	
Yes	644 (47.6)	424 (46.7)		220 (49.3)	
**Memory scores**,** Mean ± SD**	3.6 ± 2.0	3.6 ± 2.0		3.5 ± 2.0	0.647
**ADL scores**,** Median (IQR)**	0.0 (0.0, 0.0)	0.0 (0.0, 0.0)		0.0 (0.0, 0.0)	0.626
**IADL scores**,** Mean ± SD**	0.3 ± 0.8	0.3 ± 0.7		0.3 ± 0.8	0.141
**Disability**,** n (%)**					0.842
No	817 (60.4)	546 (60.2)		271 (60.8)	
Yes	536 (39.6)	361 (39.8)		175 (39.2)	
**Fall down**,** n (%)**					0.635
No	1116 (82.5)	745 (82.1)		371 (83.2)	
Yes	237 (17.5)	162 (17.9)		75 (16.8)	
**Hip fracture**,** n (%)**					0.410
No	1338 (98.9)	895 (98.7)		443 (99.3)	
Yes	15 (1.1)	12 (1.3)		3 (0.7)	
**Edentulism**,** n (%)**					0.501
No	1081 (79.9)	720 (79.4)		361 (80.9)	
Yes	272 (20.1)	187 (20.6)		85 (19.1)	
**The total number of chronic diseases**,** n (%)**					0.476
1	189 (14.0)	130 (14.3)		59 (13.2)	
2	331 (24.5)	224 (24.7)		107 (24)	
3	283 (20.9)	197 (21.7)		86 (19.3)	
≥4	550 (40.7)	356 (39.3)		194 (43.5)	

*Cfsfp* Children’s financial support for parents, *Pfsfc* Parents’ financial support for their children, *LOS,* Length of Hospital Stay, *IADL* Instrumental Activities of Daily Living, *ADL* Activity of Daily Living

### Feature selection

**Fig. 2:**
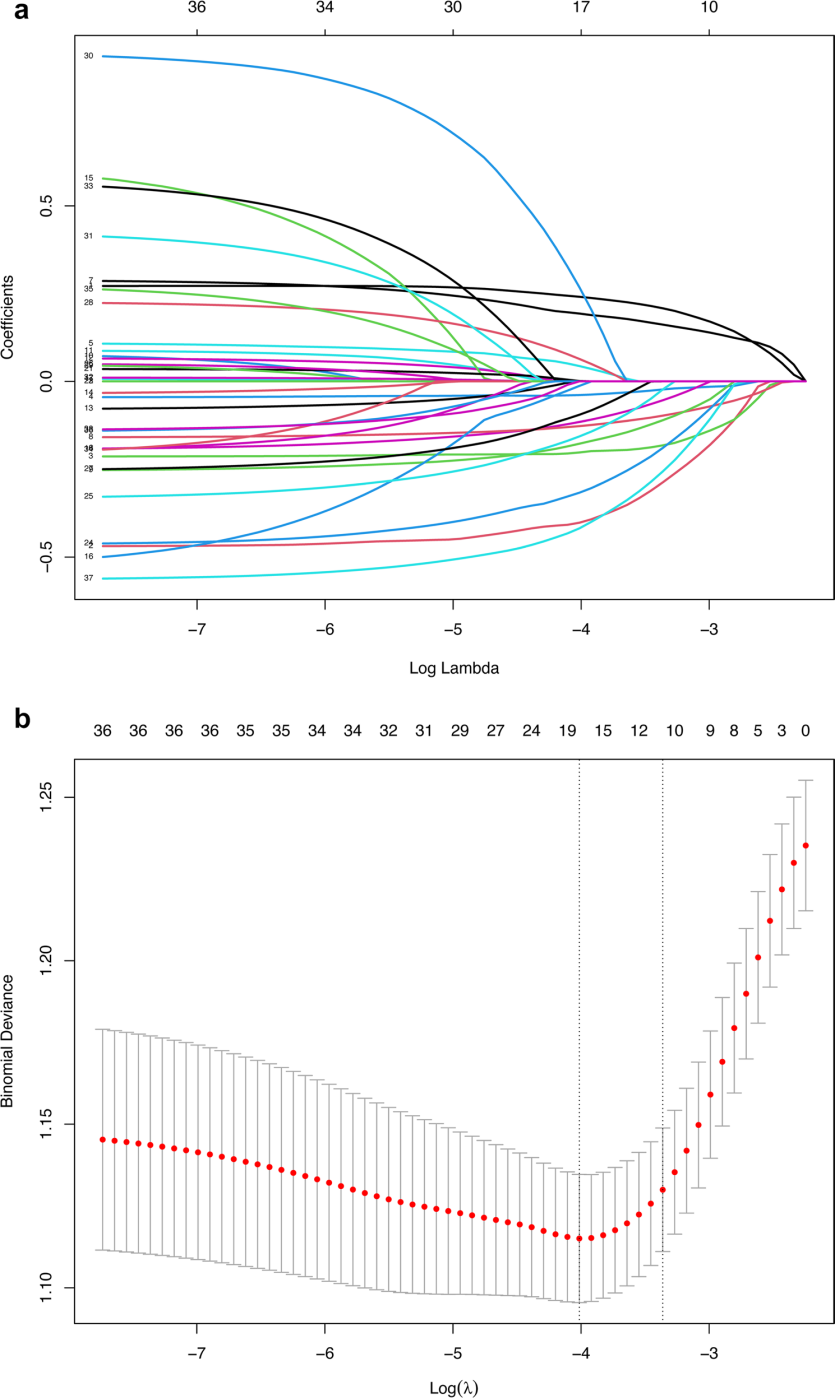
The results of the LASSO regression analysis. **a** The coefficient trajectories of all 36 variables across different regularization parameters. **b** The cross-validation curves (10-fold cross-validation), where the left vertical dashed line indicates the λ.min value and the right vertical dashed line corresponds to λ.1se. LASSO, Least Absolute Shrinkage and Selection Operator

We employed LASSO regression for variable selection, using 10-fold cross-validation to tune the model. Figure 2a illustrates the detailed variable screening process, where each curve corresponds to the shrinkage trajectory of a single variable. Notably, variables with greater importance exhibit a later convergence to a coefficient of 0 during the regularization process. In the visualization presented in Fig. 2b, two distinct dashed lines are clearly visible. These lines respectively represent two key tuning parameters for LASSO, λ.min and λ.1se, each holding specific significance for model optimization. To mitigate the potential risk of overfitting, λ.1se was selected as the candidate optimal λ value. When λ.1se was adopted, the model incorporated 10 variables, striking a favorable balance between model parsimony (simplicity) and robust predictive performance. Thus, we selected λ.1se as the optimal value for this study. Following LASSO selection, the 10 candidate variables were: ADL scores, IADL scores, memory scores, gender, self‑reported health, sleep duration, sleep quality, life satisfaction, education, and retire. To assess multicollinearity, VIF analysis was calculated for these variables. All retained features showed VIF < 5, confirming acceptable independence for inclusion in subsequent modeling (Supplementary Table 1). Thus, the final set of predictors consisted of the same 10 variables identified by LASSO. These were used as inputs for the subsequent machine‑learning model development.

### Evaluation of model performance

**Fig. 3:**
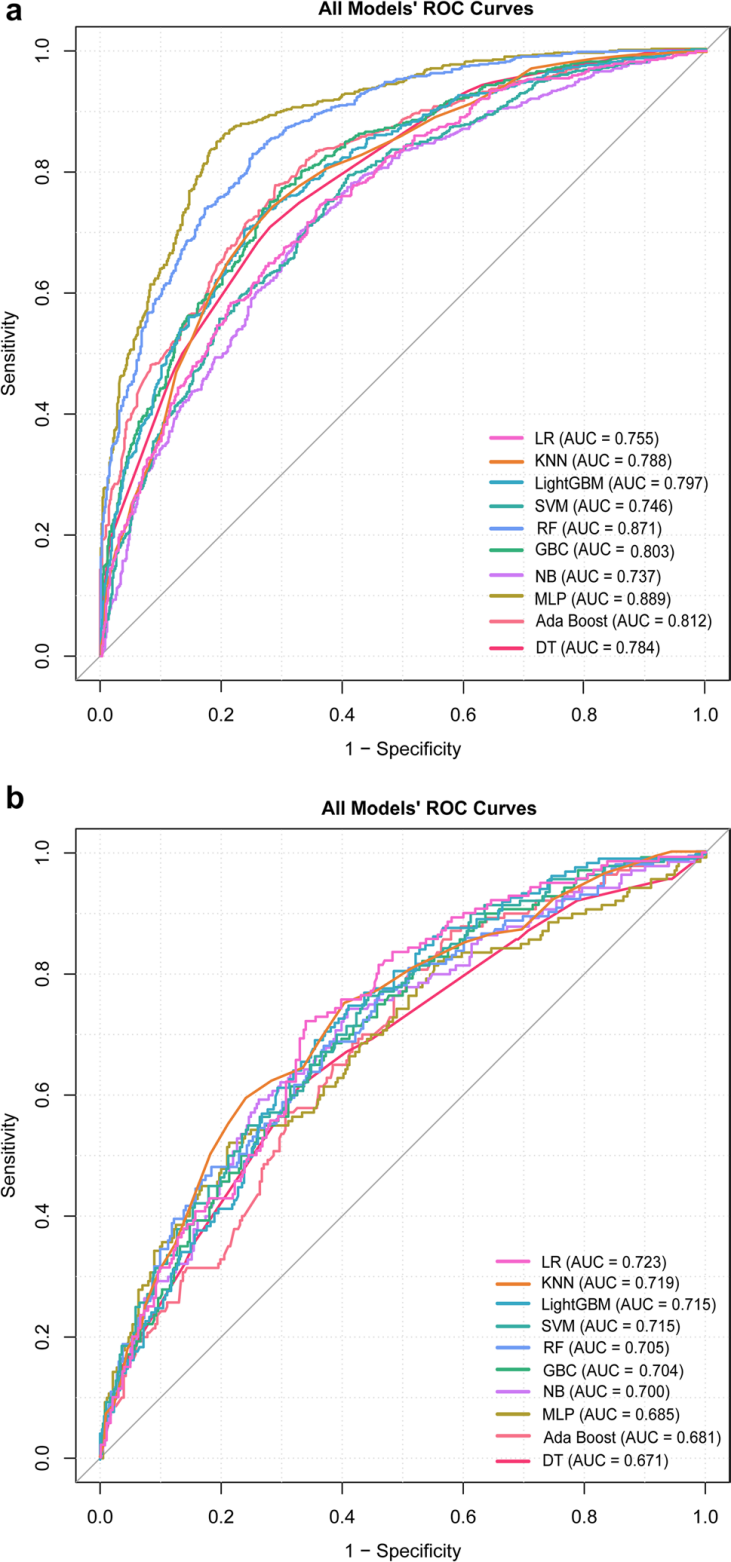
ROC curves for the ten machine learning models in the training set (**a**) and testing set (**b**). LR, Logistic Regression; MLP, Multilayer Perceptron; KNN, k-Nearest Neighbors; NB, Naive Bayes; GBC, Gradient Boosting Classifier; LightGBM, Light Gradient Boosting Machine; Ada Boost, Adaptive Boosting; SVM, Support Vector Machine; RF, Random Forest; DT, Decision Tree

Ten ML models were constructed and assessed on both training and test datasets. The corresponding ROC curves are presented in Fig. 3a (training) and Fig. 3b (testing). Comprehensive performance metrics are provided in Tables 3 and 4. During training, the MLP model achieved the highest AUC (0.889), followed by the RF model (AUC = 0.871), AdaBoost model (AUC = 0.812), GBC model (AUC = 0.803), LightGBM model (AUC = 0.797), KNN model (AUC = 0.788), DT model (AUC = 0.784), LR model (AUC = 0.755), SVM model (AUC = 0.746), and NB model (AUC = 0.737). When evaluated on the test set, the LR model exhibited the strongest discriminative performance with an AUC of 0.723 (95% CI: 0.674–0.772), outperforming the KNN model (AUC = 0.719), LightGBM model (AUC = 0.715), SVM model (AUC = 0.715), RF model (AUC = 0.705), GBC model (AUC = 0.704), NB model (AUC = 0.700), MLP model (AUC = 0.685), AdaBoost model (AUC = 0.681), and DT model (AUC = 0.671). The LR model also demonstrated higher sensitivity (0.657) and F1-score (0.553) compared to the other nine models, whereas NB recorded the best accuracy (0.694), precision (0.510), and specificity (0.759).

**Table 3:** Comparison of the predictive ability of several models in the training set

Model	AUC	Cutoff	Accuracy	Sensitivity	Specificity	Precision	F1
**LR**	0.755	0.360	0.684	0.683	0.685	0.684	0.684
**KNN**	0.788	0.380	0.730	0.743	0.717	0.724	0.733
**LightGBM**	0.797	0.440	0.731	0.736	0.725	0.728	0.732
**SVM**	0.746	0	0.670	0.659	0.680	0.673	0.666
**RF**	0.871	0.420	0.779	0.789	0.768	0.773	0.781
**GBC**	0.803	0.430	0.730	0.723	0.736	0.733	0.728
**NB**	0.737	0.200	0.655	0.548	0.762	0.697	0.614
**MLP**	0.889	0.620	0.818	0.810	0.825	0.822	0.816
**Ada Boost**	0.812	0.500	0.735	0.741	0.728	0.732	0.736
**DT**	0.784	0.330	0.714	0.707	0.720	0.717	0.712

*LR* Logistic Regression, *MLP* Multilayer Perceptron, *KNN* k-Nearest Neighbors, *NB* Naive Bayes, *GBC* Gradient Boosting Classifier, *LightGBM* Light Gradient Boosting Machine, *Ada Boost* Adaptive Boosting, *SVM* Support Vector Machine, *RF* Random Forest, *DT* Decision Tree

**Table 4:** Comparison of the predictive power of several models in the testing set

Model	AUC	Cutoff	Accuracy	Sensitivity	Specificity	Precision	F1
**LR**	0.723	0.360	0.667	0.657	0.671	0.477	0.553
**KNN**	0.719	0.380	0.658	0.643	0.664	0.466	0.541
**LightGBM**	0.715	0.440	0.667	0.643	0.678	0.476	0.547
**SVM**	0.715	0	0.649	0.629	0.658	0.456	0.529
**RF**	0.705	0.420	0.649	0.636	0.655	0.456	0.531
**GBC**	0.704	0.430	0.647	0.621	0.658	0.453	0.524
**NB**	0.700	0.200	0.694	0.550	0.759	0.510	0.529
**MLP**	0.685	0.620	0.631	0.571	0.658	0.432	0.492
**Ada Boost**	0.681	0.500	0.631	0.521	0.681	0.427	0.469
**DT**	0.671	0.330	0.644	0.629	0.651	0.451	0.525

*LR* Logistic Regression, *MLP* Multilayer Perceptron, *KNN* k-Nearest Neighbors, *NB* Naive Bayes, *GBC* Gradient Boosting Classifier, *LightGBM* Light Gradient Boosting Machine, *Ada Boost* Adaptive Boosting, *SVM* Support Vector Machine, *RF* Random Forest, *DT* Decision Tree

The prediction thresholds (cut-off values) reported in Tables 3 and 4 were derived by maximizing Youden’s index (sensitivity + specificity – 1) on the training set. In clinical practice, the choice of an optimal threshold can be adapted to specific scenarios—for example, a lower threshold may be favored in screening contexts to enhance sensitivity and capture more at-risk individuals, whereas a higher threshold could be used in resource-limited settings to improve specificity and prioritize those at greatest risk. This flexibility increases the model’s utility across diverse clinical and public‑health scenarios.

To statistically compare discriminative performance, pairwise DeLong tests were conducted on the test‑set AUCs of all ten models. The results confirmed that the LR model’s AUC was significantly superior to that of most other models (all *p* < 0.05; see Supplementary Table 2 for complete pairwise comparisons). The optimized hyperparameters of all models are summarized in Supplementary Table 3.

**Fig. 4:**
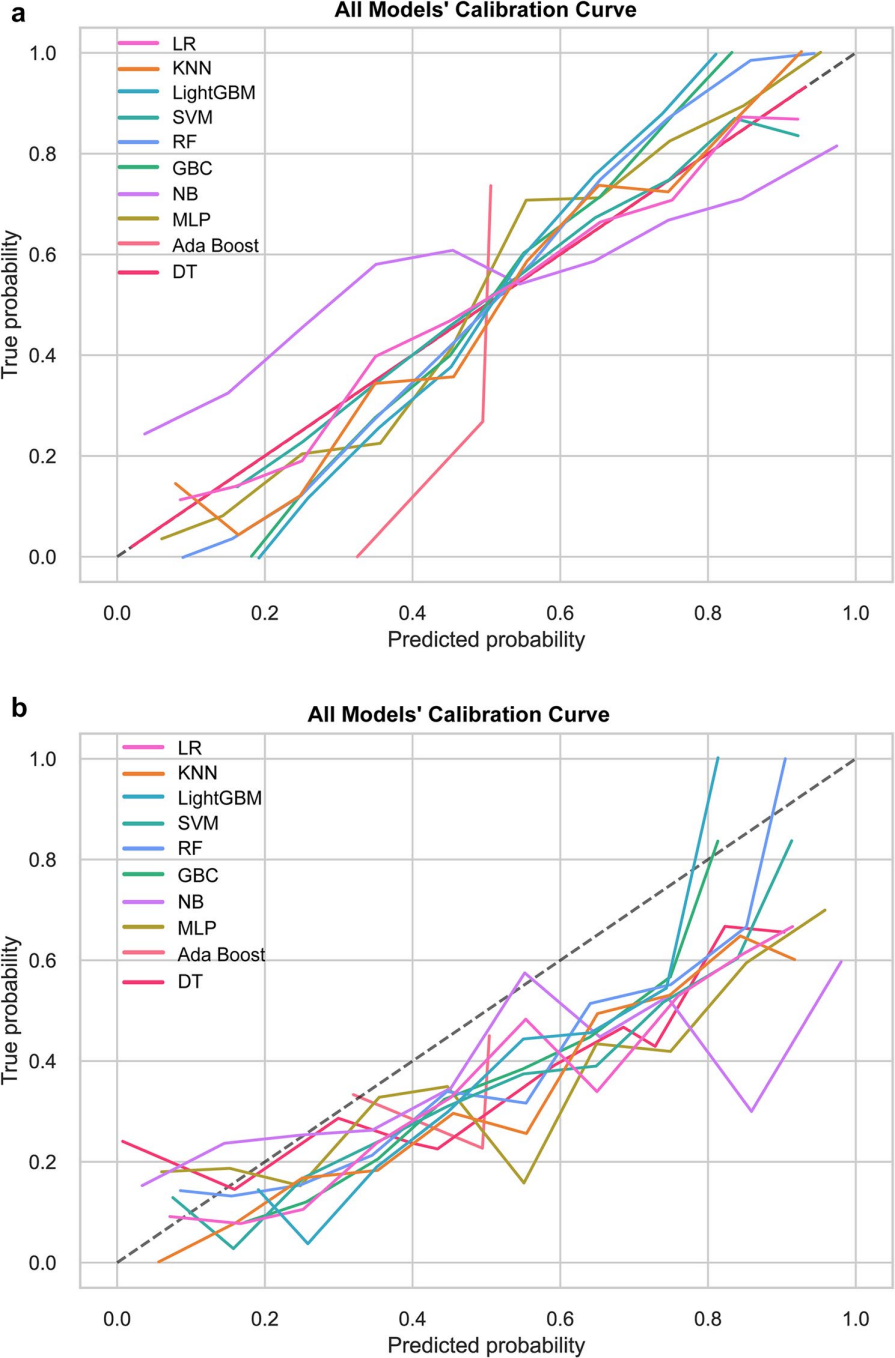
Calibration curves for the ten machine learning models in the training set (**a**) and testing set (**b**). LR, Logistic Regression; MLP, Multilayer Perceptron; KNN, k-Nearest Neighbors; NB, Naive Bayes; GBC, Gradient Boosting Classifier; LightGBM, Light Gradient Boosting Machine; Ada Boost, Adaptive Boosting; SVM, Support Vector Machine; RF, Random Forest; DT, Decision Tree

**Fig. 5:**
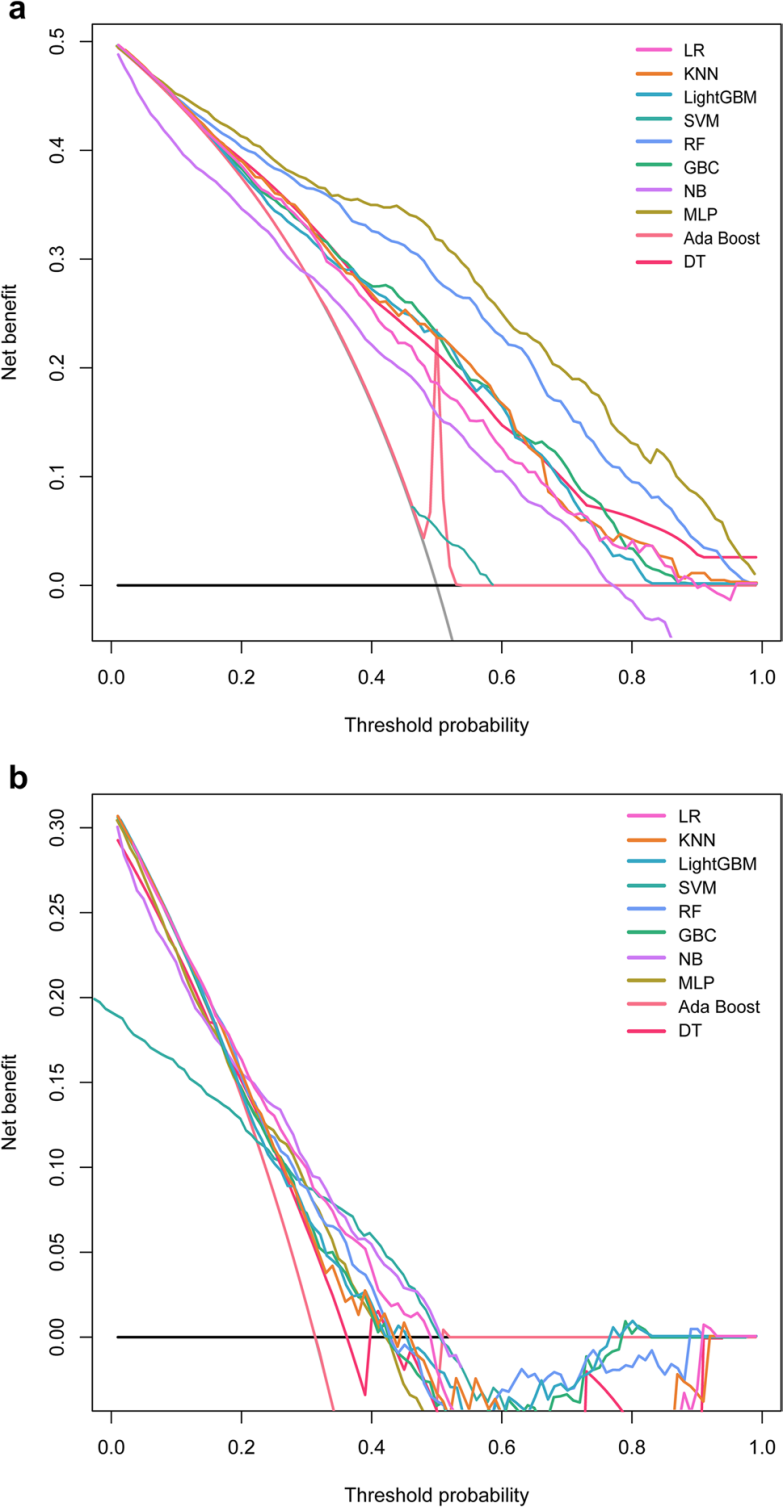
Decision curves for the ten machine learning models in the training set (**a**) and testing set (**b**). LR, Logistic Regression; MLP, Multilayer Perceptron; KNN, k-Nearest Neighbors; NB, Naive Bayes; GBC, Gradient Boosting Classifier; LightGBM, Light Gradient Boosting Machine; Ada Boost, Adaptive Boosting; SVM, Support Vector Machine; RF, Random Forest; DT, Decision Tree

Calibration curves in Fig. 4a (training) and 4b (testing) showed that the LR model’s calibration curve was most aligned with the ideal curve, indicating a superior correspondence between predicted probabilities and actual observed event rates. DCA results (Fig. 5a and b) showed that the LightGBM model offered the highest net benefit across threshold probabilities. Despite a moderate net benefit, the LR model exhibited consistent performance in both training and testing phases. Based on this comprehensive assessment, we selected the LR model for subsequent analysis.

### Evaluation of the importance of variables

**Fig. 6:**
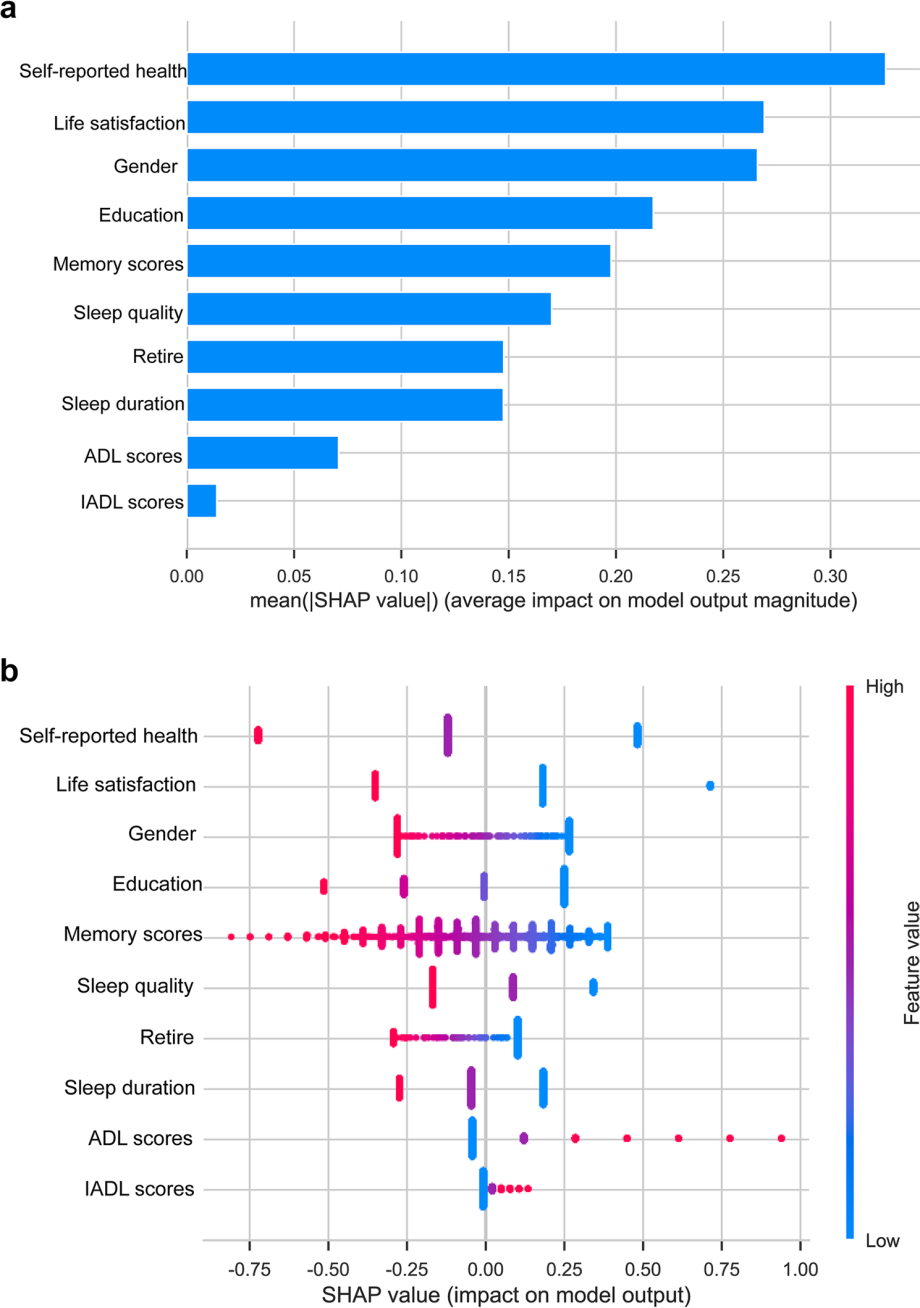
Feature importance for depression in older patients with gastrointestinal or chronic liver diseases, as determined by the LR model. (**a**) Beeswarm plot illustrating SHAP values for each predictive variable and demonstrating their association with depression. (**b**) The mean absolute SHAP value of each feature illustrates SHAP feature importance. LR, Logistic Regression; SHAP, Shapley Additive exPlanations

We assessed feature importance in the LR model through the SHAP framework and present the results in Fig. 6. SHAP values quantify the magnitude and direction of each variable’s impact on predictions, where larger absolute values reflect more substantial influence [42]. It should be noted that SHAP values reflect associative contributions within the model’s logic and are not intended to imply direct biological or causal relationships. Nonetheless, they offer valuable insight into model decision-making and help identify clinically relevant predictors.

Figure 6a shows variables ordered from top to bottom in ascending order of their contribution to depression prediction, with the vertical axis defined by an SHAP value of 0. Red points to the right of this axis indicate a positive contribution to the predicted outcome (i.e., increased depression risk). In contrast, blue points to the right of the axis indicated a negative contribution (i.e., decreased depression risk). Figure 6b displays the ranking of predictor variables based on their mean absolute SHAP values, with feature importance declining consistently from the top to the bottom of the chart. The five most influential factors for predicting depression in this cohort were: self-reported health > life satisfaction > gender > education > memory scores, all of which were negatively correlated with the occurrence of depression. For example, individuals who rated their self-reported health as “good” exhibited negative SHAP values, suggesting a lower likelihood of depression. Similarly, those who rated their life satisfaction as “good” also had negative SHAP values, indicating a reduced risk of depression. Additionally, female participants showed greater susceptibility to depression. Individuals with less education showed an increased risk of depression, whereas those with better memory performance had lower odds of depression.

### Clinical application of models

**Fig. 7:**
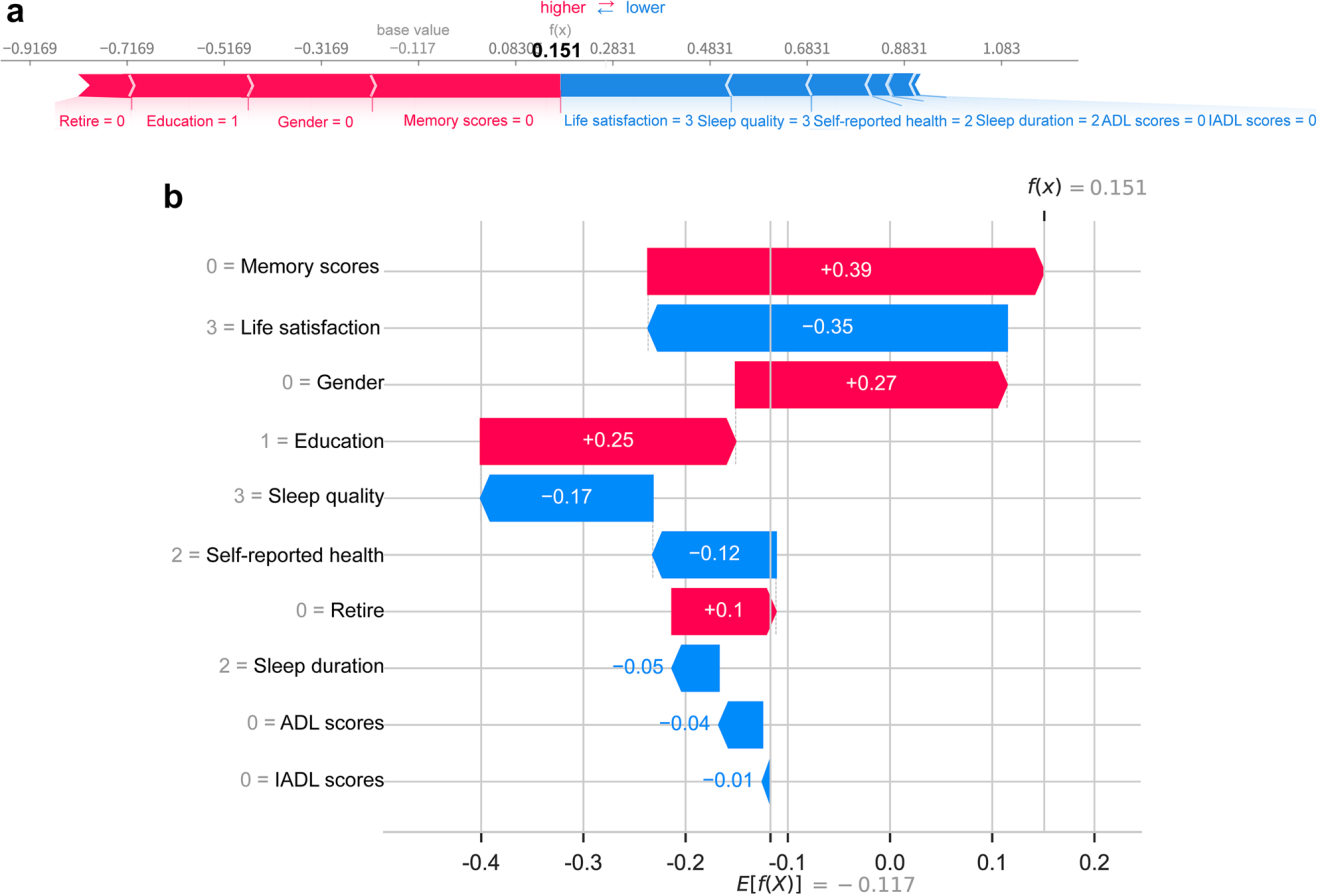
SHAP individual prediction visualization plots. **a** The force plot displays the contribution breakdown of each feature, indicating both the direction and magnitude of its effect. **b** The waterfall plot illustrates the cumulative contribution of each variable in moving from the baseline prediction to the final output for this representative case. SHAP, Shapley Additive exPlanations

To further elaborate on how the model predicts outcomes for individual patients, a single participant was randomly chosen, and a visualization was generated to illustrate the LR model’s prediction for this individual (Fig. 7a). In this plot, red indicators represent positive contributions to the prediction (increased risk of depression), while blue indicators represent negative contributions (decreased risk of depression); the f(x) value corresponds to the actual SHAP value for each factor. For this individual, the LR model predicted a higher risk of depression relative to the baseline. The key factors driving this prediction, based on their SHAP values, were memory scores, life satisfaction, gender, education, and sleep quality. Figure 7b displays a waterfall plot, which details the specific contribution values of these variables to the LR model’s prediction of depression risk.

### Correlation matrix of variables

Supplementary Fig. 2 illustrates the correlations among the 10 key variables. Specifically, IADL and ADL scores were negatively correlated with self-reported health. Sleep duration was inversely related to sleep quality, while memory scores and retire were positively associated with education.

## Discussion

The elevated prevalence of depression among patients with GID or CLD [43, 44] underscores the clinical importance of early and precise risk prediction. To address this unmet clinical demand, the present study leveraged the extensive CHARLS database to explore the application of ML techniques, a promising yet under-investigated approach in this context, to predict depression and provide a translatable tool for clinicians managing elderly patients with GID or CLD. Furthermore, we developed ten ML models (LR, MLP, SVM, KNN, LightGBM, GBC, RF, NB, AdaBoost, and DT). And we systematically evaluated their predictive performance and decision-making capabilities to identify the optimal model for clinical application. The LR model demonstrated the strongest performance on the testing set, achieving an AUC of 0.723 (95% CI: 0.674–0.772), an accuracy of 0.667, a sensitivity of 0.657, a specificity of 0.667, and an F1-score of 0.553. In clinical diagnosis, an AUC greater than 0.7 indicates that a model can effectively distinguish between individuals with and without a condition, suggesting reliability [45]. Thus, the LR model developed in this study can be considered a clinically useful tool for assessing depression risk among older adults affected by GID or CLD.

SHAP-based interpretation identified the five key predictors of depression: self-reported health, life satisfaction, gender, education and memory scores. These insights provide actionable information to support early detection of depression in clinical practice. Additionally, LASSO regression screened 10 key variables, and subsequent SHAP interpretability analysis identified potential risk factors for depression, including female gender, lower educational attainment, reduced memory scores, non-retired status, and elevated ADL and IADL scores. In contrast, factors associated with a reduced risk of depression included greater life satisfaction, positive self-reported health, improved sleep quality, and longer sleep duration. These protective relationships are consistently supported by existing literature.

Self-reported poor health status often indicates a pessimistic perception of personal well-being. Such subjective appraisal can contribute to helplessness, reduce the capacity to cope with stress, and ultimately elevate the risk of depression [46]. Conversely, higher life satisfaction is associated with an 8% to 46% lower risk of various adverse health outcomes, including depression, mortality, limited physical function, chronic pain, sleep disturbance episodes, reduced physical activity frequency, and mental health issues (e.g., negative affect, loneliness, and hopelessness) [47]. Gender differences represent a well-documented risk factor for depression in the literature: older women face a 1.597-fold higher risk of depression compared to older men [48]. This disparity may be attributed to biological mechanisms, including differences in ovarian hormone regulation [49]. In rural China, older women often lose their traditional role as homemakers with advancing age; particularly following spousal loss, they frequently face social isolation and diminished family support networks, which further elevates the likelihood of depressive symptoms [50]. Additionally, cross-sectional data from a multinational study on late-life depression (encompassing 18 countries) revealed that in the United States, adults with less education showed more severe depressive symptoms compared to those with more education [51]. The observed association between lower educational attainment and depression may be mediated by structural disadvantages, including income instability and suboptimal job quality, that engender chronic economic stress, which in turn exerts deleterious impacts on mental well-being [52, 53].

Moreover, a previous systematic review and meta-analysis demonstrated that depression is strongly associated with deficits in multiple cognitive domains, including working and long-term memory [54]. Consistent with prior research in Chinese populations, depression correlates with reduced memory function among older adults [55], a result strongly supported by our analysis. Beyond cognitive performance, depression also exhibits a strong connection to sleep quality. Extensive research indicates that sleep problems are linked to elevated risk of depressive symptoms [56]. Specifically, people experiencing poor sleep often report higher levels of anxiety, depressed mood, and irritability. A bidirectional interaction exists between sleep and depression: inadequate sleep may predispose individuals to depression, and depressive states can further impair sleep [57]. Furthermore, short sleep duration has been scientifically validated as a significant risk factor for depression [58]. From a mechanistic perspective, insomnia is associated with dysregulation of the noradrenergic system, which is a key modulator of emotional memory, characterized by either hyperactivation or deficient inhibitory control [59]. Moreover, orexin (also known as hypocretin) plays a significant role in mood regulation. Elevated cerebrospinal fluid orexin A levels observed in certain depressed patients may lead to overactivation of this system, further disrupting sleep-wake cycles and exacerbating insomnia [60].

The study further demonstrated that non-retired patients showed higher rates of depression, suggesting that the social role transition into retirement significantly shapes mental health outcomes. For non-retired patients, the transition to retirement may induce feelings of loss and reduced social engagement, both of which may increase their risk of developing depression [61]. Furthermore, impairments in ADL and IADL force individuals to rely on others to meet their basic daily needs. This reliance not only directly erodes individuals’ sense of autonomous control but also precipitates a decline in self-esteem. Additionally, it can foster doubts regarding personal capabilities and the meaning of life, ultimately exacerbating depressive symptoms [62–64].

### Limitations and strengths

Several limitations of this study warrant consideration. First, depression symptom screening relied on self-reported data from the CES-D-10, which introduces potential biases. Recall bias may occur if individuals underestimate transient depressive symptoms, whereas social desirability bias may cause underreporting due to mental health stigma. While validation studies have confirmed that the CES-D-10 aligns with clinical depression diagnoses, it is not equivalent to a formal psychiatric assessment conducted using DSM-5 criteria. This limitation may result in the misclassification of subclinical depression cases. Second, the exclusion of relevant psychosocial factors may limit the comprehensiveness of the predictive models. Third, the lack of an external validation set, a key element for assessing model robustness and generalizability in clinical research, restricts the evaluation of broader applicability. Fourth, since the data were derived exclusively from Chinese patients, the model’s performance in other populations remains unverified. Future research should incorporate multi-regional and demographically diverse cohorts to improve generalizability. Finally, although this study adopted a longitudinal design to predict incident depression using baseline predictors, it did not account for potential time-varying effects of these predictors. All predictors were assessed only at baseline, and their changes over the follow-up period were not incorporated into the models. Future studies with more frequent assessments could integrate time-updated covariates to better capture dynamic risk profiles over time.

Despite these limitations, the study offers several notable strengths. First, the CHARLS database used in this research is a high-quality, large-scale cohort dataset specific to China, which enhances the reliability and accuracy of the model by ensuring sufficient sample size and representative demographic characteristics. Second, the application of LASSO regression improved feature selection efficiency. This method not only avoided model overfitting but also preserved clinical interpretability by identifying only 10 key predictive features. These features are clinically accessible and thus have broad applicability in clinical settings. The LR model constructed in this analysis showed moderate discriminative ability (AUC = 0.723). Lastly, we used the SHAP framework to build an interpretable model, which allows users to intuitively understand the mechanisms by which the model predicts depression risk in elderly Chinese patients with GID or CLD.

Based on the results and limitations of this investigation, subsequent studies could focus on addressing current methodological constraints and expanding the scope of inquiry. First, future investigations should enroll larger and more demographically diverse cohorts, including participants from different geographic regions, with varying socioeconomic backgrounds, and across a broader spectrum of GID or CLD severities. Enrolling such diverse cohorts will strengthen the model’s applicability to heterogeneous clinical populations. Second, recent advances in AI have enabled the use of sophisticated deep learning methods, such as convolutional and recurrent neural networks (CNNs and RNNs), which have demonstrated strong potential for enhancing prediction accuracy [65, 66]. Medical imaging tools also present promising approaches for improving model performance [67]. These technologies may enable the integration of multidimensional data (e.g., electronic health record-derived clinical metrics, imaging data, or wearable device-generated physiological signals) that were not considered in this study, which may enhance both the precision and detail of depression risk assessments in this population. Future studies should investigate the potential of these advanced AI approaches to develop more comprehensive and robust predictive frameworks. Finally, future research should prioritize verifying the real-world clinical utility of the LR model identified in this study through well-designed randomized controlled trials (RCTs). Such trials could evaluate whether the implementation of the LR model in clinical practice is effective. For example, guiding targeted depression screening or early intervention in elderly patients with GID or CLD leads to meaningful improvements in patient-centered outcomes, such as reduced depression incidence, enhanced treatment adherence, or improved quality of life. RCT-based validation will also provide critical evidence to support the integration of this model into routine clinical workflows.

## Conclusions

In this study, we developed and compared ten ML algorithms to build predictive models for depression in older Chinese adults with GID or CLD, based on clinically available measures from the CHARLS database. Among these, the LR model performed best, showing good discriminative capability and stability. The SHAP-based interpretable predictive model clarified the key factors driving the risk of depression, providing a reliable tool for clinical practice. Further research could aim to evaluate the generalizability of the LR model across various healthcare environments to facilitate its integration into standard clinical practice.

## Supplementary Information

Supplementary Material 1.

Supplementary Material 2.

Supplementary Material 3.

Supplementary Material 4.

Supplementary Material 5.

## Data Availability

The dataset supporting the findings of this investigation is publicly accessible and can be retrieved directly from the official CHARLS portal at [https://charls.charlsdata.com/](https:/charls.charlsdata.com) .
