# Supplementary material for: A longitudinal cohort study: developing an interpretable machine learning model to predict incident depression risk in elderly Chinese patients with gastrointestinal or chronic liver diseases
Source: BMC Geriatr. 2026 Feb 25;26:563. doi: 10.1186/s12877-026-07239-7 (PMC13101395; doi:10.1186/s12877-026-07239-7)
Supplement: Supplementary file 3 — Supplementary Material 3. [file 12877_2026_7239_MOESM3_ESM.docx]

| **Variables** | **Variance Inflation Factor** |
| --- | --- |
| **Life satisfaction (Poor)** | 1.012 |
| **Self-reported health (Poor)** | 1.020 |
| **Sleep quality (Fair)** | 1.022 |
| **Self-reported health (Fair)** | 1.023 |
| **Life satisfaction (Good)** | 1.027 |
| **Life satisfaction (Fair)** | 1.028 |
| **Sleep duration (Normal sleep)** | 1.030 |
| **Education (Elementary school graduate)** | 1.037 |
| **Sleep duration (Long sleep)** | 1.041 |
| **Self-reported health (Good)** | 1.051 |
| **Education (Middle school graduate)** | 1.054 |
| **Memory scores** | 1.067 |
| **Gender (Female)** | 1.068 |
| **Gender (Male)** | 1.068 |
| **Sleep quality (Poor)** | 1.069 |
| **Education (High school graduate and above)** | 1.101 |
| **Sleep duration (Short sleep)** | 1.105 |
| **Sleep quality (Good)** | 1.113 |
| **Education (Below elementary school)** | 1.140 |
| **Retire (No)** | 1.208 |
| **Retire (Yes)** | 1.208 |
| **ADL scores** | 1.229 |
| **IADL scores** | 1.231 |

**Supplementary Table 1. Variance inflation factor analysis for predictors retained after LASSO selection.**

IADL, Instrumental Activities of Daily Living; ADL, Activity of Daily Living
