# Supplementary material for: A longitudinal cohort study: developing an interpretable machine learning model to predict incident depression risk in elderly Chinese patients with gastrointestinal or chronic liver diseases
Source: BMC Geriatr. 2026 Feb 25;26:563. doi: 10.1186/s12877-026-07239-7 (PMC13101395; doi:10.1186/s12877-026-07239-7)
Supplement: Supplementary file 4 — Supplementary Material 4. [file 12877_2026_7239_MOESM4_ESM.docx]

| **Model** | **LR** | **KNN** | **LightGBM** | **SVM** | **RF** | **GBC** | **NB** | **MLP** | **Ada Boost** | **DT** |  |  |
| --- | --- | --- | --- | --- | --- | --- | --- | --- | --- | --- | --- | --- |
| **LR** | NA | 0.035 | 0.028 | 0.022 | 0.009 | 0.007 | 0.005 | 0.001 | 0.001 | <0.001 |  |  |
| **KNN** | 0.035 | NA | 0.162 | 0.149 | 0.043 | 0.039 | 0.008 | 0.002 | 0.002 | <0.001 |  |  |
| **LightGBM** | | | 0.028 | 0.162 | NA | 0.987 | 0.041 | 0.037 | 0.006 | 0.001 | 0.001 | <0.001 |
| **SVM** | 0.022 | 0.149 | 0.987 | NA | 0.099 | 0.008 | 0.003 | 0.001 | 0.001 | <0.001 |  |  |
| **RF** | 0.009 | 0.043 | 0.041 | 0.009 | NA | 0.175 | 0.036 | 0.004 | 0.003 | 0.001 |  |  |
| **GBC** | 0.007 | 0.039 | 0.037 | 0.008 | 0.175 | NA | 0.034 | 0.005 | 0.004 | 0.001 |  |  |
| **NB** | 0.005 | 0.008 | 0.006 | 0.003 | 0.036 | 0.034 | NA | 0.029 | 0.027 | 0.006 |  |  |
| **MLP** | | 0.001 | 0.002 | 0.001 | 0.001 | 0.004 | 0.005 | 0.029 | NA | 0.181 | 0.007 |  |
| **Ada Boost** | 0.001 | 0.002 | 0.001 | 0.001 | 0.003 | 0.004 | 0.027 | 0.181 | NA | 0.006 |  |  |
| **DT** | <0.001 | <0.001 | <0.001 | <0.001 | 0.001 | 0.001 | 0.006 | 0.007 | 0.006 | NA |  |  |

**Supplementary Table 2. Pairwise DeLong Test P-values for AUC Comparisons Among the Nine Machine Learning Models.**

NA (Not Applicable) appears for model self-comparisons along the diagonal, as well as for pairwise comparisons where the AUC values were identical or the ROC curves were numerically indistinguishable, rendering DeLong’s test non-informative.

LR, Logistic Regression; MLP, Multilayer Perceptron; KNN, k-Nearest Neighbors; NB, Naive Bayes; GBC, Gradient Boosting Classifier; LightGBM, Light Gradient Boosting Machine; Ada Boost, Adaptive Boosting; SVM, Support Vector Machine; RF, Random Forest; DT, Decision Tree.
